# Knee loading stimulates cortical bone formation in murine femurs

**DOI:** 10.1186/1471-2474-7-73

**Published:** 2006-09-19

**Authors:** Ping Zhang, Min Su, Shigeo M Tanaka, Hiroki Yokota

**Affiliations:** 1 Departments of Biomedical Engineering, Indiana University – Purdue University Indianapolis, Fesler Hall 115, 1120 South Drive, Indianapolis IN 46202, USA; 2 Department of Anatomy and Cell Biology, Indiana University School of Medicine, Fesler Hall 115, 1120 South Drive, Indianapolis IN 46202, USA; 3 Graduate School of Natural Science and Technology, Kanazawa University, Ishikawa, Japan

## Abstract

**Background:**

Bone alters its architecture and mass in response to the mechanical environment, and thus varying loading modalities have been examined for studying load-driven bone formation. The current study aimed to evaluate the anabolic effects of knee loading on diaphyseal cortical bone in the femur.

**Methods:**

Using a custom-made piezoelectric loader, 0.5-N loads were laterally applied to the left knee of C57/BL/6 mice at 5, 10, 15, and 20 Hz for 3 minutes per day for 3 consecutive days. Animals were sacrificed for examination 13 days after the last loading. The contralateral femur was used as a non-loading control, and the statistical significance of loading effects was evaluated with *p *< 0.05.

**Results:**

Although diaphyseal strains were measured as small as 12 μstrains, bone histomorphometry clearly demonstrated frequency-dependent enhancement of bone formation. Compared to a non-loading control, bone formation on the periosteal surface was significantly enhanced. The loading at 15 Hz was most effective in elevating the mineralizing surface (1.7 x; *p *< 0.05), mineral apposition rate (1.4 x; *p *< 0.001), and bone formation rate (2.4 x; *p *< 0.01). The loading at 10 Hz elevated the mineralizing surface (1.4 x; *p *< 0.05), mineral apposition rate (1.3 x; *p *< 0.01), and bone formation rate (1.8 x; *p *< 0.05). The cross-sectional cortical area and the cortical thickness in the femoral diaphysis were significantly increased by loading at 10 Hz (both 9%) and 15 Hz (12% and 13%, respectively).

**Conclusion:**

The results support the anabolic effects of knee loading on diaphyseal cortical bone in the femur with small *in situ *strain, and they extend our knowledge on the interplay between bone and joints. Strengthening the femur contributes to preventing femoral fractures, and the discovery about the described knee loading might provide a novel strategy to strengthen osteoporotic bones. Further analyses are required to understand the biophysical and molecular mechanism behind knee loading.

## Background

Osteoporosis as a skeletal disorder is characterized by compromised bone strength, which leads to an increased risk of fracture. Patients with femoral fracture account for a substantial volume of orthopaedic hospitalizations. Thus, strengthening femurs is a keen healthcare issue particularly among the aging population [1]. Load-imposing exercises are recommended as a protective measure [2], but they are often limited to healthy individuals who can afford physical activities [3]. Varying loading modalities have therefore been developed in an attempt to enhance femoral bone formation, and biomechanical factors such as loading frequencies, strain and strain-induced fluid flow, and alteration in intramedullary pressure are investigated to understand load-driven anabolic responses [4-7]. Whole body vibrations, for instance, are effective in elevating bone mass in femoral trabecular bone [8]. Few mild, non-invasive loading modalities, however, have been shown to stimulate formation of cortical bone in the femur. In this report, one form of joint loading – knee loading – is described as a potential means to provoke anabolic responses in femoral cortical bone.

Joint loading is a relatively new form of mechanical stimulus in which loads are laterally applied to the epiphyseal bone. Long bones such as an ulna and a femur are apparently less stiff in the lateral direction than in the axial direction, and the epiphysis rich in trabecular bone is softer than the diaphysis composed of cortical bone. Therefore, our rationale behind joint loading is that exposing the least rigid part of bone – epiphyseal bone – to lateral loads might effectively trigger load-driven anabolic responses in the diaphysis. In fact, elbow loading in our previous mouse study [9,10] revealed enhanced cortical bone formation in the ulnar diaphysis. Compared to an axial ulna-bending modality [11], elbow loading required at most one fourth of the loads (0.5-N forces) to induce bone formation with the same loading timetable. The current study extends elbow loading to knee loading and examines the anabolic effects on femoral cortical bone.

We have previously shown that knee loading stimulates bone formation in the tibia [12,13], but no study has been performed to evaluate its effects on the femur. A knee is anatomically a complex structure and application of loads to the knee does not imply that the same effects are expected to the tibia and the femur. With knee loading, loads are applied to the lateral and medial epicondyles of the femur as well as the lateral and medial condyles of the tibia. Since the tibial condyle is positioned closer to the loader than the femoral epocondyle, the femoral epiphysis is expected to receive fewer loads than the tibial epiphysis. In addition, anabolic responses to knee loading at different frequencies may vary between the tibia and the femur. If knee loading is effective in the femur, it provides a possibility in future to prevent bone loss throughout the femur including the femoral neck. A substantial bone loss or damage to the femoral neck could result in a serious healthcare problem particularly in the aging population.

Two specific questions addressed herein are (1) whether lateral loads applied to the epiphysis enhance cortical bone formation in the femoral diaphysis; and (2) if so, whether bone formation is dependent on loading frequencies. Using femurs *ex vivo*, we previously showed that molecular transport in diaphyseal lacunae was activated with knee loading [14]. However, any *in vivo *consequences or potential mechanisms of enhanced molecular transport were not examined. In this study, we address the question of whether loads applied to the joint site would enhance bone formation in the remote diaphyseal bone.

To answer that question we used a custom-made piezoelectric mechanical loader for knee loading. Dependence of loading frequencies on anabolic responses has been pointed out in varying loading studies [11,15], and we employed loading frequencies at 5, 10, 15 and 20 Hz. The results in this study clearly support that knee loading can effectively enhance bone formation in a loading frequency-dependent manner.

## Methods

### Experimental animals

Sixty-one female C57/BL/6 mice ~14 weeks of age (body weight of ~20 g) were used (Harlan Sprague-Dawley, Inc., Indianapolis, IN). Five animals were housed per cage at the Laboratory Animal Resource Center of Indiana University School of Medicine, and they were fed with standard laboratory chow and water *ad libitum*. The animals were allowed to acclimate for 2 weeks before the experiment. All procedures, performed in this study, were in accordance with the Institutional Animal Care and Use Committee guidelines at Indiana University School of Medicine and approved.

### Mechanical loading

**Figure 1:**
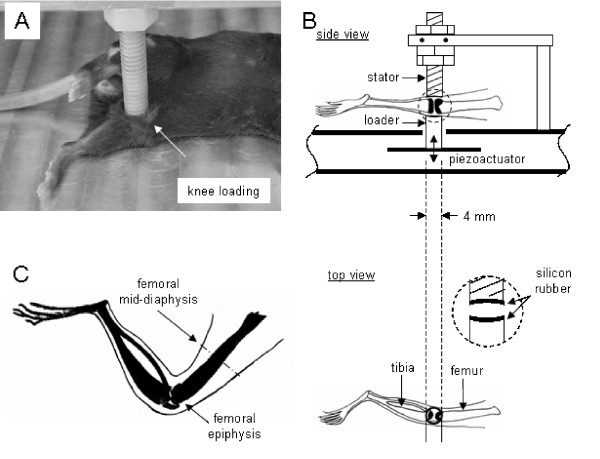
Setup of a mechanical loader for knee loading used in this study. (A) Custom-made piezoelectric mechanical loader for knee loading. (B) Schematic diagram illustrating the loading site. The knee is placed between the stator and the loader (4 mm in diameter). (C) Hindlimb showing the femoral mid-diaphysis (site for a bone histomorphometric analysis).

The mouse was placed in an anesthetic induction-chamber to induce sedation and mask-anesthetized using 2% isoflurane. Using the custom-made piezoelectric mechanical loader, mechanical loads were applied for 3 minutes per day for 3 consecutive days to the left knee through the lateral-medial direction (Fig. 1). The mice were randomly divided into four groups for four loading frequencies (5, 10, 15 or 20 Hz, N = 8), and the loads with a peak-to-peak force of 0.5 N were applied. Using the same procedure, the right knee was placed under the loader without oscillatory loading and used as the non-loading control. After loading, the mouse was allowed normal cage activity and any abnormal behavior, weight loss or a diminished food intake was monitored.

### Piezoelectric joint loader

The loader consisted of four bimorph-type piezoelectric actuators (LPD 1260X; Megacera, Saitama, Japan). A voltage signal was sent through a 16-bit data-acquisition board (PCI-6052E; National Instruments, Austin, TX) and a piezo-driver (model PZD 700-L; Trek, New York, NY) (Fig. 1). Before the loading experiment, loads applied to the knee were calibrated using an anesthetized mouse. A strain gauge (CEA-06-062UW; Vishay Measurements Group, Raleigh, NC) was attached on the aluminum cantilever connected to the stator and known forces from 0 to 10 N were given. To position the knee properly, the lower end of the loading rod and the upper end of the supporter were designed to form a pair of semispherical cups. The lateral and medial epicondyles of the femur together with the lateral and medial condyles of the tibia were confined in the cups. Because of the complex anatomy of the knee, the lateral side of the femur is less projected outwards. Therefore, the femoral epicondyle apparently receives fewer loads than the tibial condyle. The tip of the loader had a contact area of 4 mm diameter. To avoid stress concentration between the knee and the loader, the loading surface and the supporter were covered with silicon rubber.

### Measurement of strain

**Figure 2:**
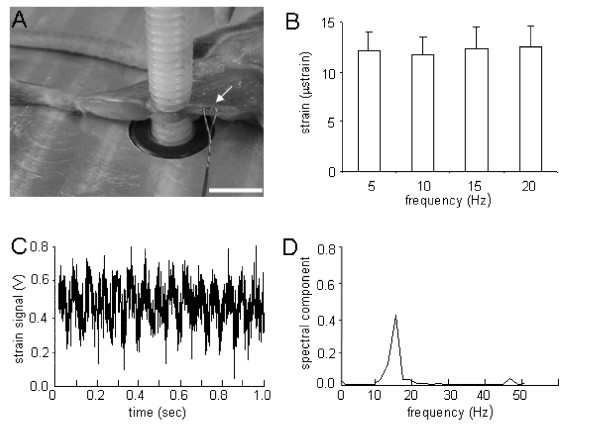
Strain measurement with knee loading. (A) Mouse femoral diaphysis showing a strain gauge attached at the midpoint in the femur. Scale bar = 5 mm. (B) Measured strains in response to loads of 0.5 N forces applied to the knee with the loading frequency at 5, 10, 15 and 20 Hz. The results are expressed as mean ± SEM. (C) Representative strain signal in a time domain in response to knee loading at 15 Hz. (D) Strain signal in a frequency domain in response to loads at 15 Hz.

Five animals were used for strain measurements (Fig. 2). After euthanasia, the periosteal surface of a left femur was exposed. A strain gauge (Model EA-06-015DJ-120, Measurements Group Inc., NC) was trimmed into 0.5 mm in width and 2.2 mm in length, and attached with glue to the mid-diaphysis (50% along the length of the femur). Note that it was unavoidable to remove a part of surrounding tissue for attachment of the strain gauge but the loading site in the knee was kept intact. Bone histomorphometry was conducted using 0.5-N loads, and therefore the strain was also measured with the same loading conditions. The distal epiphysis was loaded at 5, 10, 15 and 20 Hz with 0.5 N. Voltage signals from the strain gauge were processed with a signal conditioning amplifier (2210, Measurement Group Inc., NC), and Fourier transform was conducted. The measurement was repeated 5 times, and the peak-to-peak voltage was converted to the actual strain value using the standard calibration line [15,16]. Loads were confined with the loading rod, and no bending moment was presumably generated to the femur or the tibia.

### Fluorescence labeling and sample preparation

**Figure 3:**
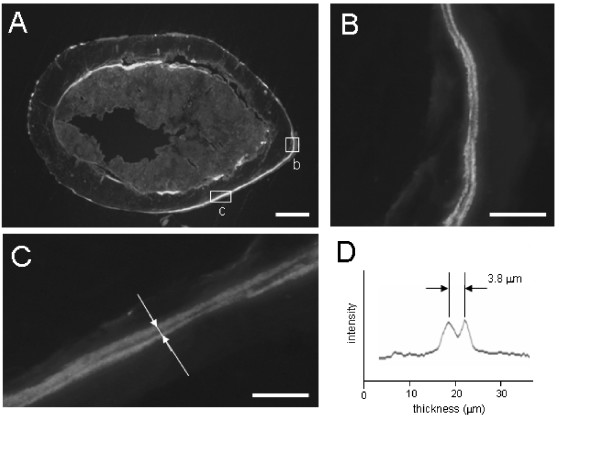
Femoral cross-sections with knee loading. (A) Cross-section of the loaded femur with 0.5 N forces at 15 Hz. The section was obtained from the mid-diaphysis. Scale bar = 200 μm. (B) & (C) Double-labeled periosteal surface of the loaded femur. Bright lines represent fluorescent calcein strips. Scale bar = 20 μm. (D) Fluorescence intensity along the line indicated in C. The distance between two lines, 3.8 μm in the diagram, indicates the newly formed bone in 4 days after 3 min loading per day for 3 consecutive days.

Mice were given an intraperitoneal injection of calcein (Sigma, St. Louis, MO), a fluorochrome dye, at 30 μg/g body mass on days 2 and 6 after the last loading (Fig. 3). Animals were sacrificed 13 days after the last loading, and femurs were harvested for histomorphometric analyses. The isolated bones were cleaned of soft tissues, and the distal and proximal ends were cleaved to allow infiltration of the fixatives with 10% neutral buffered formalin. After 48 h in the fixatives, bones were transferred to 70% alcohol for storage. Specimens were dehydrated in a series of graded alcohols and embedded in methyl methacrylate (Aldrich Chemical Co., Milwaukee, WI). The transverse sections (~80 μm in thickness) were removed from the mid-diaphysis, ~8 mm distant from the distal end using a diamond-embedded wire saw (Delaware Diamond Knives, Wilmington, DE), and they were mounted on standard microscope slides.

### Bone histomorphometry

Using a Nikon Optiphot microscope (Nikon, Inc., Garden City, NY) and a Bioquant digitizing system (R & M Biometrics, Nashville, TN), we measured total perimeter (B.Pm), endocortical perimeter, single-labeled perimeter (sL.Pm), double-labeled perimeter (dL.Pm), and double-labeled area (dL.Ar). From these measurements we derived mineralizing surface (MS/BS = [1/2 sL.Pm + dL.Pm]/B.Pm in %), mineral apposition rate (MAR = dL.Ar/dL.Pm/4 in μm/day), and bone formation rate (BFR/BS = MAR × MS/BS × 365 in μm^3^/μm^2 ^per year). To evaluate the effects of the loading frequencies the relative parameters such as rMS/BS, rMAR, and rBFR/BS were derived as differences between the loaded and the control femurs. In drawing a fluorescent intensity curve, a MetaMorph Imaging System (version 3.6, Universal Imaging Co.) was used. Note that the fluorescent labeling data represent bone formation during the days 2 and 6 after the last loading.

The total bone cross-sectional area (mm^2^), bone medullary area (mm^2^), and cortical thickness (mm) were also measured. The cross-sectional cortical area was determined by subtracting the bone medullary area from the total bone cross-sectional area. The cortical thickness was defined as the mean distance between the endosteal and the periosteal surfaces on both anterior and posterior sides. The measurements were taken at the middle of each side, and the mean value was determined from two independent measurements. The relative alteration was calculated as differences between loaded (L) and control (C) femurs such as ([L - C]/C × 100 in %).

### Bone porosity

Intracortical porosity was determined from the tibial and femoral transverse sections of the non-loaded limbs in the mid-diaphysis (~50 μm thickness; N = 24). Using a Nikon Optiphot microscope and a Bioquant digitizing system, we measured cross-sectional cortical area (mm^2^), total porous area (mm^2^), and the number of pores whose area was larger than an identifiable threshold of 11 μm^2 ^with the optical system. From these measurements, we derived intracortical porosity (ratio of the porous area to the total bone area in %) and pore density (number/mm^2^). The mean value was calculated from 2 independent measurements.

### Microcomputed tomography (μCT)

Micro-CT was performed using a desktop μCT-20 (Scanco Medical AG, Auenring, Switzerland). The hindlimb was harvested keeping the intact knee. The sample was placed in a plastic tube filled with 70% ethanol and centered in the gantry of the machine. A series of cross-sectional images were captured in an 8-mm segment including the knee at 30-μm resolution. Images were imported into Scion Image software (Scion Corp., Fredrick, MD, USA), and three-dimensional reconstruction was conducted.

### Statistical analysis

The data were expressed as mean ± SEM. Statistical significance among groups was examined using ANOVA, and a post-hoc test was conducted using a Fisher's protected least significant difference (PLSD) for all the pairwise comparisons. A paired *t-*test was employed to evaluate statistical significance between the loaded and control femurs. All comparisons were two-tailed and statistical significance was evaluated with *p *< 0.05.

## Results

The animals used for bone histomorphometry tolerated the procedures, and any abnormal behavior including weight loss or diminished food intake was not observed. No bruising or other damages were detected at the loading site.

### In situ strain in the femoral mid-diaphysis with knee loading

In response to knee loading with 0.5 N forces, the strains at the mid-diaphysis of the femur were measured at 5, 10, 15 and 20 Hz loading frequencies (Fig. 2). The measured strains were in the range from 8 to 19 μstrain, and the mean value was ~12 μstrain regardless of the loading frequencies. No statistically significance was observed among the four loading frequencies (ANOVA, *p *= 0.99).

### Enhancement of cortical bone formation on periosteal surface

Bone formation on the periosteal surface was significantly enhanced (Table 1, Fig. 3), especially with knee loading at 10 and 15 Hz. Compared to the non-loading control, bone formation on the periosteal surface using the combined data for 4 loading groups was significantly enhanced in terms of MS/BS, MAR, and BFR/BS (1.4 x, 1.3 x, and 1.7 x, all *p *< 0.001). The loading at 10 Hz elevated the three morphometric parameters (MS/BS, MAR, and BFR/BS) by 1.4-fold (*p *< 0.05), 1.3-fold (*p *< 0.01), and 1.8-fold (*p *< 0.05) on the periosteal bone, respectively. Similarly, the loading at 15 Hz resulted in an increase in these parameters by 1.7-fold (MS/BS; *p *< 0.05), 1.4-fold (MAR; *p *< 0.001) and 2.4-fold (BFR/BS; *p *< 0.01). Unlike the periosteal surface, although bone formation on the endosteal surface was increased, no statistically significant effects were observed on the endosteal surface.

### Dependence of anabolic responses on loading frequencies

**Figure 4:**
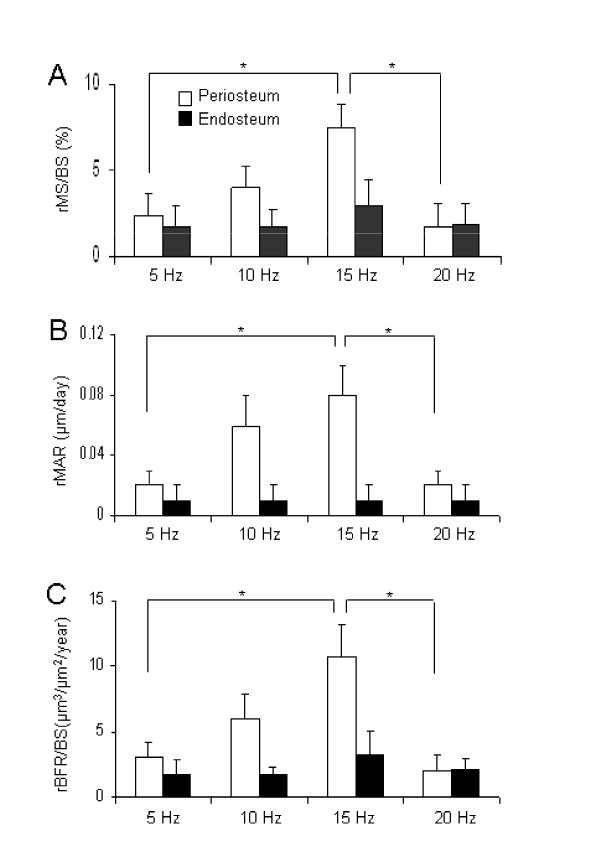
Alteration in the histomorphometric parameters on the periosteal and endosteal surfaces with knee loading at 5, 10, 15, and 20 Hz. The results are expressed as mean ± SEM. Single asterisks indicate *p *< 0.05 in Fisher's PLSD. (A) Relative MS/BS (%). (B) Relative MAR (μm/day). (C) Relative BFR/BS (μm^3^/μm^2^/year).

Dependence on the loading frequencies was also prominent in the relative parameters defined on the periosteal surface (Fig. 4). In order to further evaluate the effects of knee loading on the periosteal and endosteal surfaces, rMS/BS, rMAR, and rBFR/BS were determined. Compared to the loading at 5 Hz, the loading at 15 Hz resulted in a significant increase in three morphometric parameters (*p *< 0.01 for rMS/BS and rBFR/BS; and *p *< 0.05 for rMAR). Furthermore, compared to the loading at 20 Hz, the loading at 15 Hz resulted in an increase in these parameters (*p *< 0.01 for rMS/BS and rBFR/BS; *p *< 0.05 for rMAR). In contrast, no significant alterations were observed on the endosteal surface at any loading frequency (*p *= 0.75 – 0.99; data not shown).

### Elevation in cortical bone area and thickness

**Figure 5:**
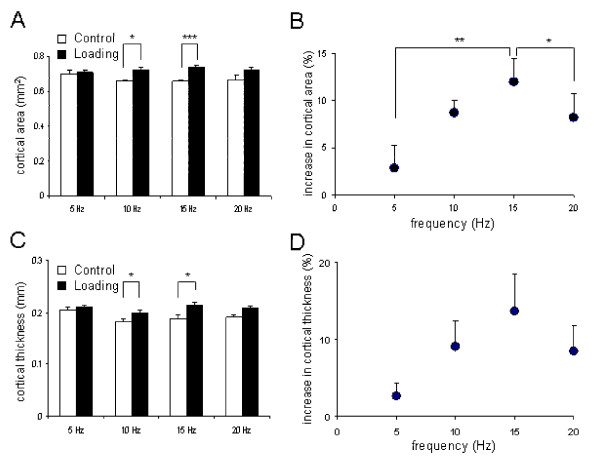
Alteration in the cross-sectional cortical area and the cortical thickness with and without knee loading at 5, 10, 15 and 20 Hz. The results are expressed as mean ± SEM. The single, double, and triple asterisks indicate *p *< 0.05, *p *< 0.01 and *p *< 0.001 in Fisher's PLSD, respectively. (A) Cross-sectional cortical area (mm^2^). (B) Increase in the cross-sectional area (% of control). (C) Cortical thickness (mm). (D) Increase in the cortical thickness (% of control).

A frequency-dependent enhancement of the cortical area and the cortical thickness was observed (Fig. 5). First, the cross-sectional cortical area was increased from 0.66 ± 0.01 mm^2 ^(control) to 0.74 ± 0.01 mm^2 ^(loading) at 15 Hz (*p *< 0.001), and from 0.66 ± 0.01 mm^2 ^(control) to 0.72 ± 0.02 mm^2 ^(loading) at 10 Hz (*p *< 0.05). No significant alteration was observed at 5 Hz (*p *= 0.43) or 20 Hz (*p *= 0.56). Second, the cortical thickness was also elevated from 0.188 ± 0.007 mm (control) to 0.213 ± 0.008 mm (knee loading) at 15 Hz (*p *< 0.05), and from 0.183 ± 0.005 mm (control) to 0.199 ± 0.005 mm (knee loading) at 10 Hz (*p *< 0.05). No significant changes were observed at 5 Hz (*p *= 0.45) or 20 Hz (*p *= 0.06). Among the four loading frequencies, the loading at 15 Hz generated a cross-sectional cortical area significantly greater than that at 5 Hz (*p *< 0.05). Regarding the cortical thickness, there was no statistical difference among the four loading frequencies (*p *= 0.19).

### Bone porosity in the tibia and the femur

**Figure 6:**
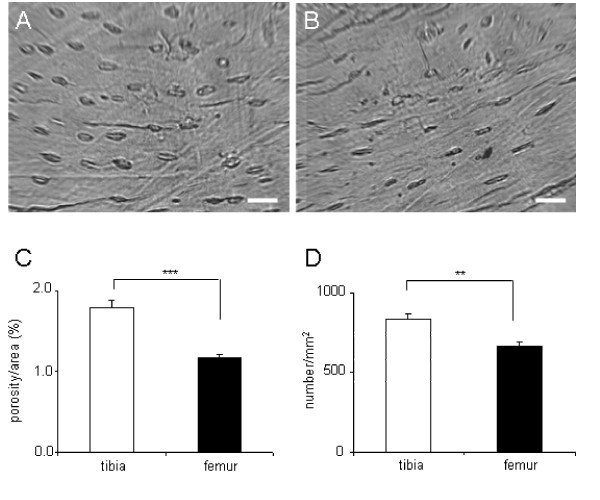
Bone porosity in the tibia and the femur. (A) Tibial histological section. The white bar is 20 μm. (B) Femoral histological section. The white bar is 20 μm. (C) Comparison of the tibia/femur porous area (%). (D) Comparison of the tibia/femur porosity density (number/mm^2^).

Bone porosity in the cortical bone was significantly different between the tibia and the femur (Fig. 6). Regarding intracortical porosity, the tibia (1.79 ± 0.10 in %) was more porous than the femur (1.18 ± 0.04) (*p *< 0.001). Similarly, the porosity density (number/mm^2^) was higher in the tibia (831 ± 42) than the femur (666 ± 20) (*p *< 0.01).

## Discussion

The histomorphometric results reveal that knee loading – lateral loads applied to the femoral distal epiphysis – increase formation of diaphyseal cortical bone in the femur. The enhancement was most significant with a 15 Hz loading frequency. Unlike a spring loading device [17], hip loading with insertion of a pair of wires [18], or femoral vein ligation [19], the described knee loading modality is non-invasive. Strain has been considered a potential determinant for load-driven bone formation [20,21], and a strain threshold required to induce anabolic responses was modeled as a function of the number of daily loading cycles [4]. With 0.5-N loads in the current study, an *in situ *strain was measured as small as 12 μstrain at the site of bone formation in the femoral mid-diaphysis. Since loads were applied 3 min per day, the number of daily loading cycles was 2,700 with the most effective loading frequency at 15 Hz. These results indicate that cortical bone formation can be enhanced with an *in situ *strain of ~10 μstrain at 1,000 – 10,000 loading cycles. Given the same loading cycles, the estimated *in situ *strain was approximately 1/10 of the predicted threshold value of 1000 μstrain [4].

**Figure 7:**
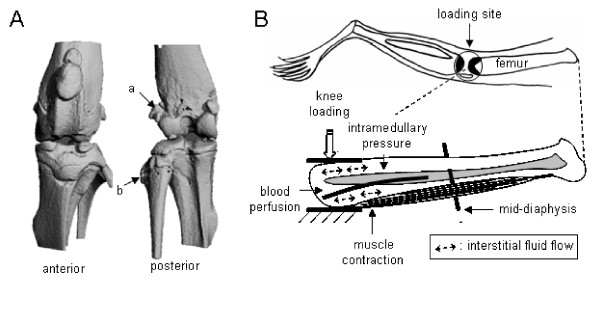
(A) Micro CT images (anterior and posterior views) of the mouse knee. The point "a" (femoral epicondyle) is located away from the loader, compared to the point "b" in the tibial side. (B) Potential contributors involved in cortical bone formation with knee loading. The illustration includes alteration in intramedullary pressure, interstitial fluid flow, muscle contraction, and blood perfusion.

Knee loading was reported as an effective means to enhance bone formation in the tibia without inducing significant *in situ *strain at the site of bone formation [12,13,22]. In the present study we demonstrated that it is effective to stimulate bone formation in the femoral diaphysis, although the most effective loading frequency differs. Many factors can contribute to the observed bone formation including load-driven blood perfusion, muscle contraction, and strain induced fluid flow (Fig. 7). One of the hypotheses is that the applied loads generate an oscillatory alteration in intramedullary pressure and the induced pressure gradient stimulates fluid flow and anabolic responses. Trabecular bone in the epiphysis is less stiff than cortical bone in the diaphysis [23,24], and Young's modulus in the lateral direction is smaller than that in the axial direction [25]. Therefore, lateral loads with knee loading to the femoral epiphysis may effectively modulate intramedullary pressure in the femoral bone cavity. Furthermore, modulation of intramedullary pressure may produce a local pressure gradient in the diaphysis and induce interstitial fluid flow on the cortical bone surface.

The results of bone formation in the current study are consistent with the reported enhancement of molecular transport *ex vivo *with knee loading [14]. Load-driven interstitial fluid flow was monitored using dye migration in histological bone sections [5] and visualized through fluid displacements in cortical and trabecular bones [26]. Recently a fluorescence-based technique, "fluorescence after photo-bleaching," was employed to evaluate molecular transport in a lacuna [14,27,28]. In order to examine the possibility of induction of molecular transport in the diaphysis with knee loading, the photo-bleaching experiments were conducted using a mouse femur *ex vivo *[14]. A fluorescently labeled lacuna in cortical bone was photo-bleached and a rate of fluorescence recovery was measured with and without loads on the epiphysis. It was reported that knee loading shortened the fluorescent recovery approximately by one quarter [14]. A further linkage analysis between alteration in intramedullary pressure and shortening of the recovery time would contribute to our understanding of the mechanism behind the anabolic responses with knee loading.

Differential sensitivity of the periosteal and endosteal surfaces was observed in response to knee loading. Although bone formation increased on both the periosteal and the endosteal surfaces, in contrast to the marked enhancement of bone formation on the periosteal surface, the endosteal surface exhibited no significant increase in any of the morphometric parameters. Our observations are consistent with some of the previous studies using other loading modalities such as tibia bending [29-31], ulna axial loading [32], and loading with a spring device [17], although strain distributions are different among loading modalities. The results may suggest a differential role of alterations in intramedullary pressure on the periosteal and endosteal surfaces, and differences in anatomical and physiological microenvironments. For instance, the non-uniform osteogenic potentials could also result from local variations in cellular and molecular compositions, and/or heterogeneity in load-driven pressure gradient and interstitial fluid flow on these two surfaces [5-7].

We examined porosity of cortical bone in the tibia and the femur as a potential cause of differential frequency dependence in response to knee loading. As reported by Qin *et al*. with their pressure experiments, we postulated that the observed frequency dependence with knee loading could be caused by interactions of microstructures in the lacunocanalicular network and intramedullary pressure [6,7]. The histological analysis revealed that cortical bone in the tibia is more porous than that in the femur. Our porosity observation indicates that femoral microstructure might be sensitive to higher frequencies that tibial microstructure. A further bone microstructures analysis would help to elucidate the role of load frequency in the mechanism of joint loading.

Limitations inherent in the present study include the restricted loading frequencies and varying numbers of daily loading cycles among the loading groups. In whole-body vibrations, it has been suggested that higher frequencies (> 30 Hz) can stimulate bone formation with a lower level of strain [33,34]. Although the anabolic responses at 20 Hz were insignificant, we do not have any information on the effects of loading frequencies above 20 Hz. Dependence of the observed anabolic responses on loading frequencies requires further investigations since a daily loading cycle and a strain rate are considered important factors, and mechanical testing should be conducted to examine strengthening of bones with knee loading. Note that although loading at 20 Hz has the highest cycle number as well as the strain rate, it did not exhibit superior capability of bone formation. We did not detect any morphological damage on loaded knees, but more careful inspection using molecular markers such as expression and activities of matrix metalloproteinases will be useful for detecting the maximum loads that do not cause any inflammation or evaluating potential regional acceleratory phenomena [35-37]. Our preliminary studies indicate that knee loading is able to enhance bone formation throughout the femur including the femoral neck, and it can stimulate healing of wounded bone. Our future analyses include the role of osteoclastic activities as well as osteoblastic activities with knee loading. A prototype human knee joint loading supporter, designed and fabricated for mechanical characterization, could be used in future for a clinical trial [38].

## Conclusion

In summary, the current mouse femur study demonstrates that knee loading is an effective means to enhance cortical bone formation on the periosteal surface in the femoral diaphysis with an *in situ *strain as small as ~10 μstrain at the site of bone formation. Furthermore, the efficacy of the observed anabolic responses was dependent on the loading frequency. The results here extend our knowledge of load-driven bone formation and the interplay between the epiphysis and the diaphysis in the femur. Strengthening femurs contributes to preventing femoral fractures, and the described knee loading might provide a novel strategy to develop mechanical therapies.

## Competing interests

The author(s) declare that they have no competing interests.

## Authors' contributions

PZ performed the animal experiments as well as bone histomorphometry and drafted the manuscript. MS carried out the strain measurements. ST conducted part of the animal experiments. HY designed the project and edited the manuscript. All authors read and approved the final manuscript.

## Pre-publication history

The pre-publication history for this paper can be accessed here:



## Figures and Tables

**Table 1:** Bone formation in periosteum and endosteum with knee loading

		MS/BS (%)	*p *value	MAR (μm/day)	*p *value	BFR/BS (μm^3^/μm^2^/year)	*p *value
5 Hz							
Periosteum	control	11.62 ± 1.18	NS	0.20 ± 0.01	NS	8.51 ± 0.84	NS
	loading	14.04 ± 1.82		0.22 ± 0.01		11.50 ± 1.67	
Endosteum	control	11.35 ± 1.80	NS	0.21 ± 0.01	NS	8.88 ± 1.53	NS
	loading	13.00 ± 2.42		0.22 ± 0.01		10.56 ± 1.98	
10 Hz							
Periosteum	control	10.60 ± 1.31	< 0.05	0.20 ± 0.01	< 0.01	7.96 ± 1.28	< 0.05
	loading	14.58 ± 1.27		0.26 ± 0.02		13.96 ± 1.75	
Endosteum	control	10.22 ± 2.89	NS	0.20 ± 0.01	NS	7.63 ± 2.25	NS
	loading	11.89 ± 2.70		0.22 ± 0.01		9.37 ± 2.31	
15 Hz							
Periosteum	control	10.61 ± 1.44	< 0.05	0.20 ± 0.01	< 0.001	7.95 ± 1.28	< 0.01
	loading	18.09 ± 2.52		0.28 ± 0.01		18.69 ± 3.13	
Endosteum	control	14.14 ± 2.15	NS	0.22 ± 0.01	NS	11.69 ± 2.03	NS
	loading	17.10 ± 2.10		0.24 ± 0.01		14.99 ± 2.23	
20 Hz							
Periosteum	control	11.81 ± 1.50	NS	0.20 ± 0.01	NS	9.04 ± 1.48	NS
	loading	13.48 ± 1.33		0.23 ± 0.01		11.06 ± 1.10	
Endosteum	control	14.17 ± 2.50	NS	0.21 ± 0.01	NS	10.55 ± 1.79	NS
	loading	15.97 ± 2.07		0.22 ± 0.02		12.67 ± 1.65	

NS indicates *p *> 0.05.
